# Supplementary material for: Reduced Tie2 in Microvascular Endothelial Cells Is Associated with Organ-Specific Adhesion Molecule Expression in Murine Health and Endotoxemia
Source: Cells. 2023 Jul 14;12(14):1850. doi: 10.3390/cells12141850 (PMC10378325; doi:10.3390/cells12141850)

Figure S1

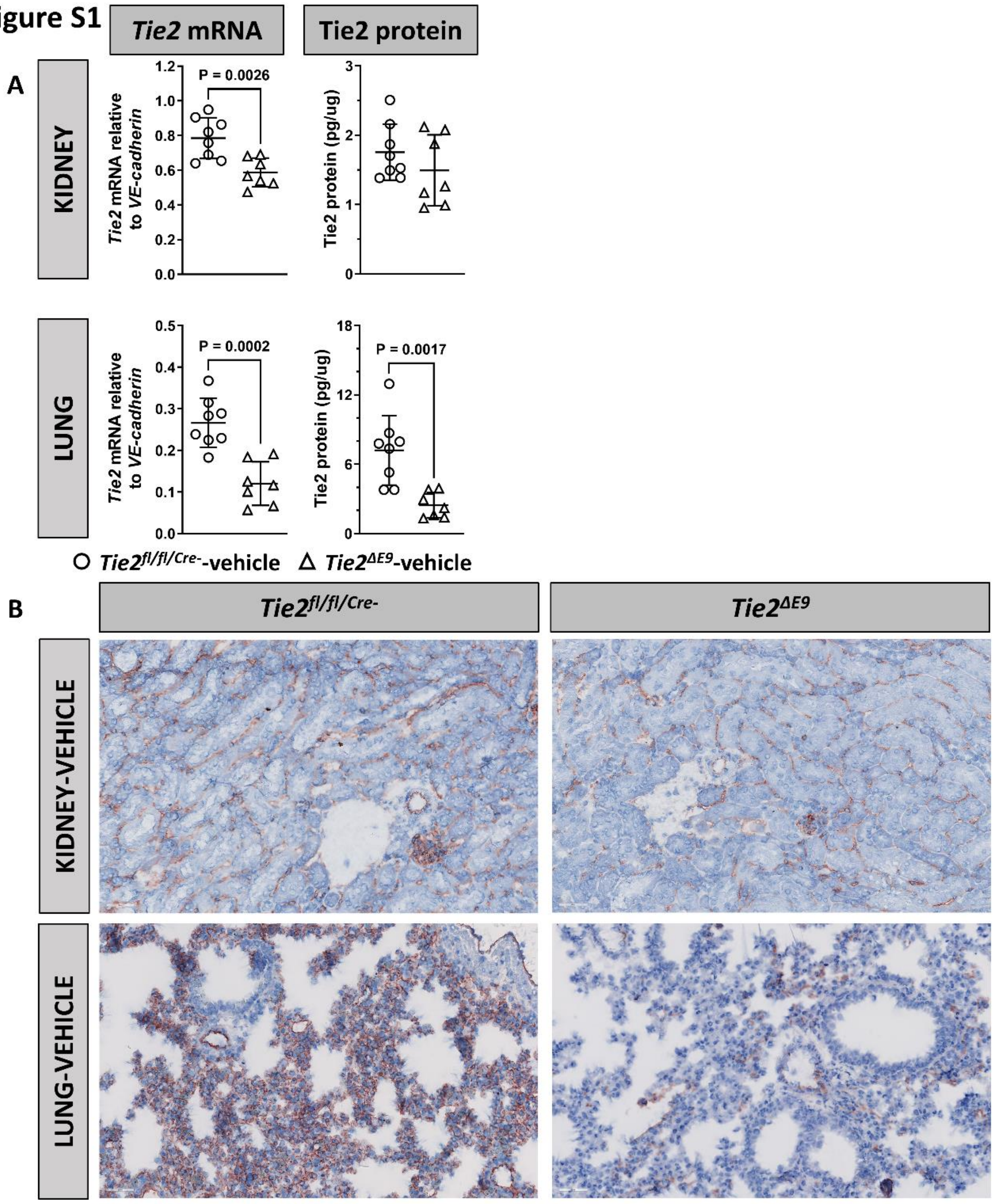

Figure S1. Tie2 mRNA and protein expression in kidney and lung. Tie2 expression levels were determined in kidney and lung of $Tie2^{fl/fl/Cre-}$ control and $Tie2^{\Delta E9}$ knock out mice after tamoxifen and vehicle treatment by (**A**) RT-qPCR (left) and ELISA (right). Graphs show individual values, and means (black lines) ± SD (error bars). Open circles: $Tie2^{fl/fl/Cre-}$-veh mice (n=8); open triangles: $Tie2^{\Delta E9}$-veh mice (n=7). Tie2 protein expression was localized by immunohistochemistry in kidney, and lung of $Tie2^{fl/fl/Cre-}$ control, and $Tie2^{\Delta E9}$ knock out mice after tamoxifen and vehicle treatment. (**B**) Photomicrographs of Tie2 staining, taken at 400× optical magnification, are shown for each genotype.

Figure S2: genotyping kidney samples, raw images

A

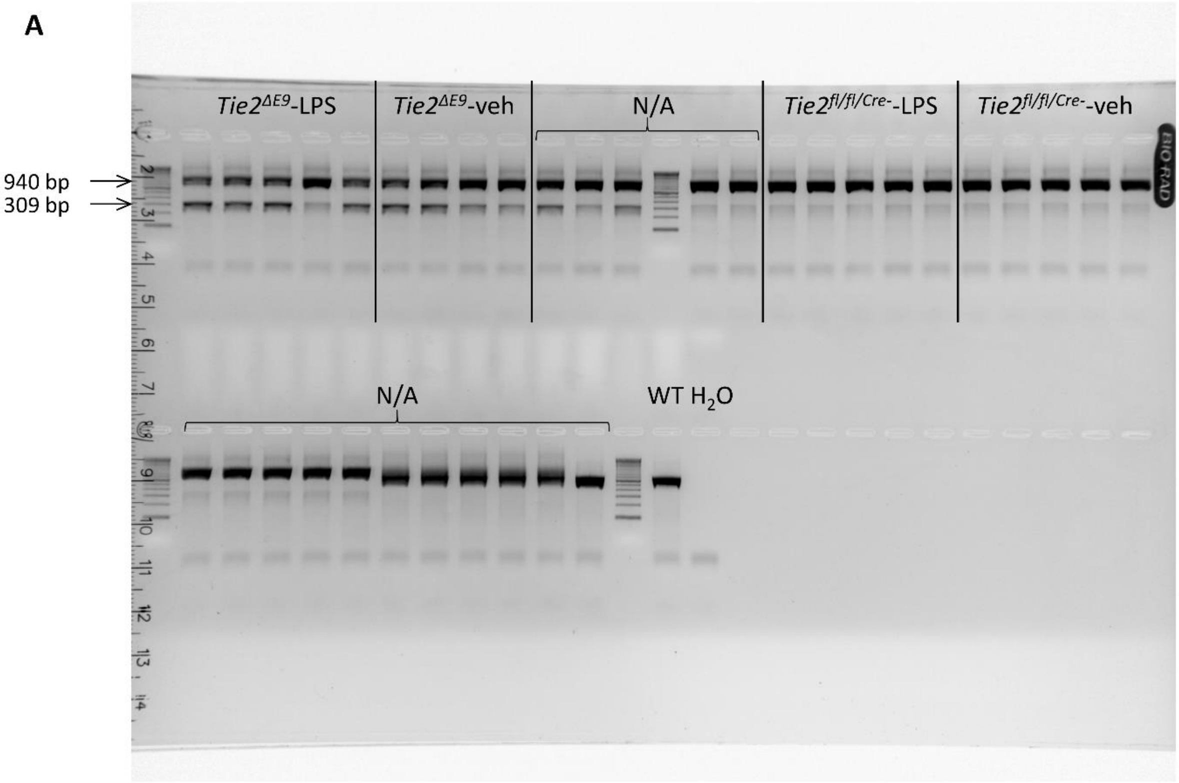

Figure S2. Genotyping organ samples, raw images. Deletion of *Tie2* exon9 by tamoxifen-induced Cre-recombinase was confirmed by genomic PCR for (**A**) kidney, (**B**) lung, and (**C**) kidney and lung. A 309 basepair (bp) PCR product indicates deletion of exon 9, a 940 bp product the presence of exon 9. Lanes represent PCR products for individual mice. N/A marked samples were not annotated

Figure S2: genotyping lung samples, raw images

B

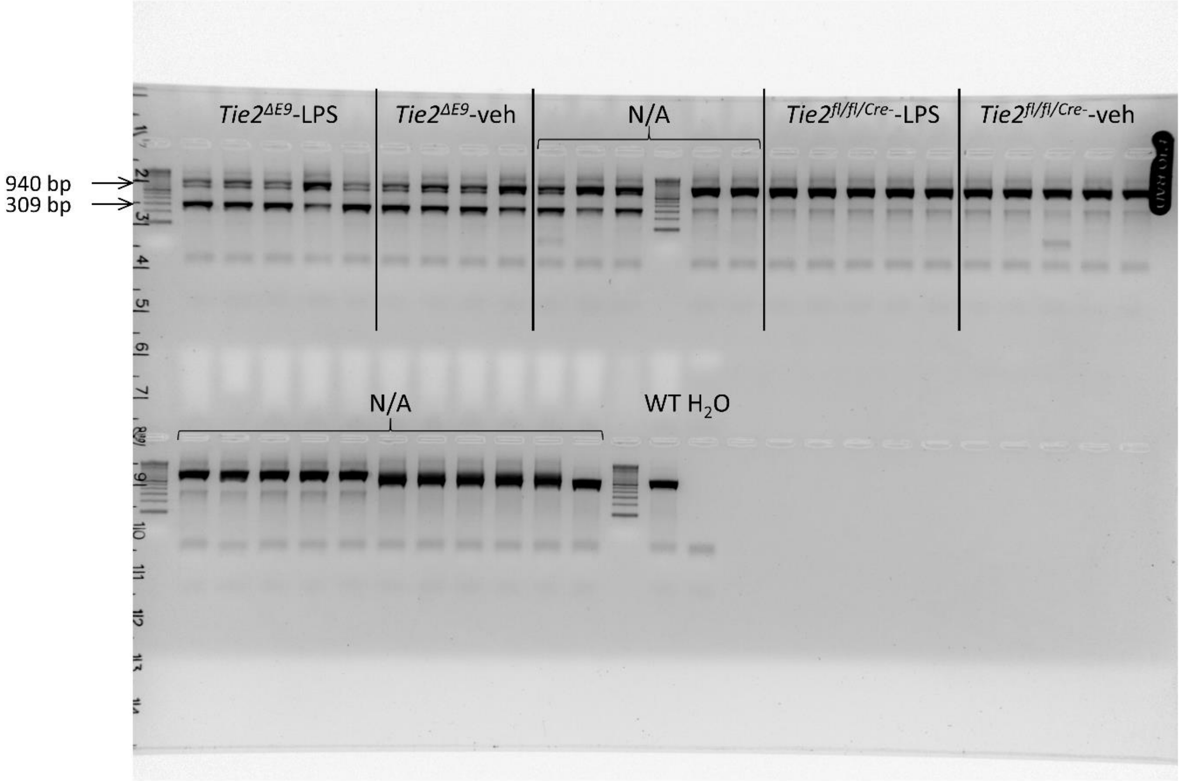

Figure S2: genotyping kidney and lung samples, raw images

C

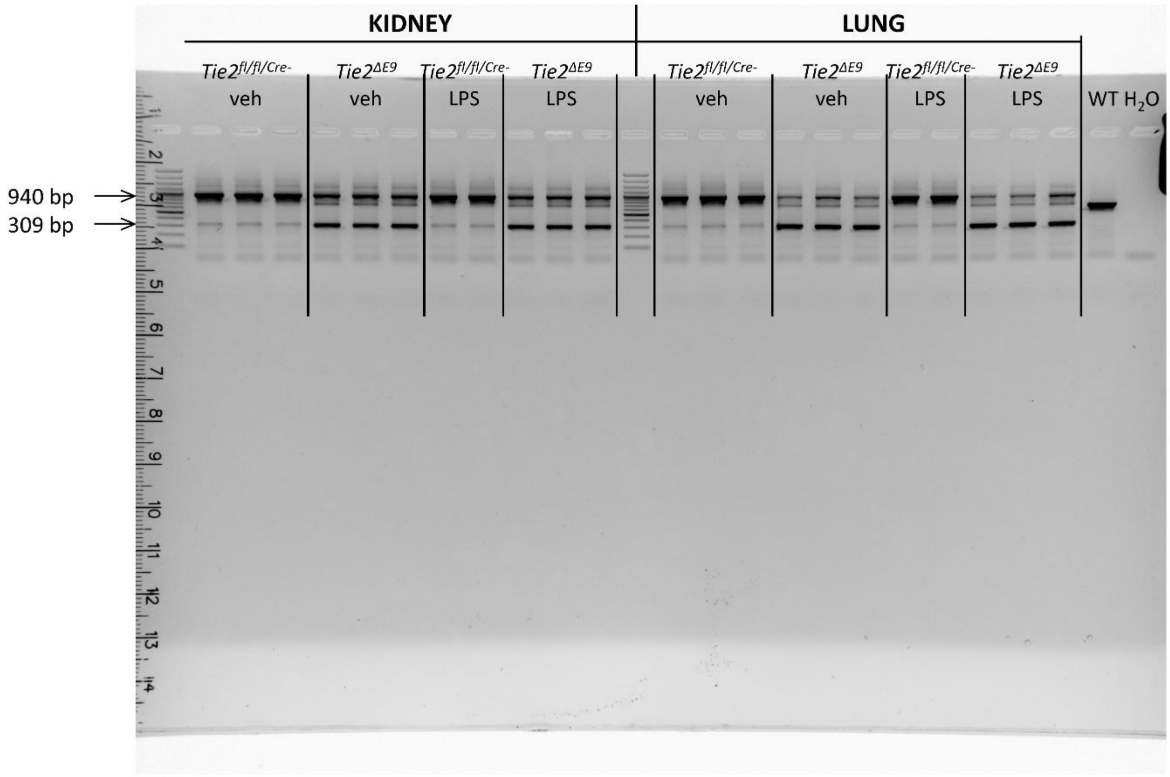

Supplement: Supplementary file 1 [file cells-12-01850-s001.zip › cells-2451593-supplementary.pdf]
